# Supplementary material for: Disruption of the HIV-1 Envelope allosteric network blocks CD4-induced rearrangements
Source: Nat Commun. 2020 Jan 24;11:520. doi: 10.1038/s41467-019-14196-w (PMC6981184; doi:10.1038/s41467-019-14196-w)
Supplement: Supplementary file 1 — Supplementary Information [file 41467_2019_14196_MOESM1_ESM.pdf]

Disruption of the HIV-1 Envelope allosteric network blocks CD4-induced rearrangements

Supplementary Tables and Figures

Henderson et al.

Supplementary Table 1: BG505 SOSIP Env Mutations

Mutation	Mutations
F1	V68I, S115V, A204L, V208L, V255W, N377L, M426W, M434W, H66S
F2	V68I, S115V, A204L, V208L, V255W, H66S
F3	V68I, A204V, V208L, V255L, H66S
F4	V68I, S115V, V208L, V255L, H66S
F5	V68I, S115V, A204L, V208L, V255W, N377L, H66S
F6	V68I, S115V, A204L, V208L, V255L, W69L
F7	V68I, S115V, A204L, V208L, V255L, W69V
F8	V68I, S115V, A204L, V255L, V208L, W69A
F9	V68I, S115V, A204L, V208L, V255W, N377L, M426W, H66S
F10	V68I, S115V, A204L, V208L, V255W, N377L, M434W, H66S
F11	V68I, S115V, A204V, V208L, V255L, H72P, H66S
F12	V68I, S115V, V208L, V255L, H66K
F13	V68I, S115V, A204L, V208L, V255W, N377L, M426W, M434W
F14	V68I, A204V, V208L, V255L
F15	V68I, A204L, V208L, V255W, N377L
Vt1	Y177F, T320L, D180A, Q422L, Y435F, Q203M, E381L, R298M, N302L, N300L
Vt2	Y177F, T320L, D180A, Q422L, Y435F, Q203M, N302L, N300L
Vt3	Y177F, T320L, D180A, Q422L, Y435F, Q203M, R298M, N302L, N300L
Vt4	Y177F, T320L, D180A, Q422L, Y435F, Q203M, E381L, N302L, N300L
Vt5	T320L, D180A, Q422L, Q203M, E381L, R298M, N302L, N300L
Vt6	T320L, D180A, Q422L, Q203M, N302L, N300L
*Vt7**	I201C, A433C, L154M, N300M, N302M, T320L
Vt8	T320M, Q422M, Q203M, N302L, N300L
Vt9	T320M, Q422M, Q203M, N302L, N300L, S174V

* Design DS-SOSIP.4mut from [13]

Supplementary Table 2: CD4 Binding Kinetics and Affinities

CD4 Binding*	k_a ($M^{-1}s^{-1}$)	k_d (s^{-1})	K_d (nM)
**BG505**	1.12E+04 ± 5.77E+03	6.10E-04 ± 9.50E-05	67.9 ± 26.4
**BG505 F14**	1.72E+04 ± 4.75E+03	1.13E-03 ± 1.00E-04	73.0 ± 26.2
**BG505 Vt8**	9.44E+03 ± 6.65E+02	7.81E-04 ± 6.40E-05	83.9 ± 12.6
**BG505 F14/Vt8**	2.92E+04 ± 5.35E+03	4.52E-04 ± 7.40E-05	15.6 ± 0.4

* Values reported as the average of two replicate measures ± the standard error

Supplementary Table 3: bnAb Binding Affinities (K_d [nM])

	BG505	BG505 F14	BG505 Vt8	BG505 F14/Vt8
**PGT145**	64.2	76.52	68.04	80.2
**PGT151**	26.9	23.34	24.04	24.04
**VRC01**	611.7	565.7	268.9	313.7
**PG9**	444	45.9	335	336.8
**VRC26**	67.6	50.23	60.4	50.6
**PGT121**	222.3	218.6	260.3	262

* Values reported from fit to the average of two replicate measures

Supplementary Table S4: Cryo-EM Data Collection and Refinement Statistics

	BG505 F14 SOSIP	BG505 F14/Vt8 SOSIP
Data Collection		
Microscope	FEI Titan Krios	FEI Titan Krios
Voltage (kV)	300	300
Electron dose ($e^-/Å^2$)	42	42
Detector	Falcon 3	Falcon 3
Pixel Size (Å)	1.08	1.08
Defocus Range (μm)	~1.5-3	~1.5-3
Magnification	75000	75000
Reconstruction		
Software	cisTEM	cisTEM
Particles	77632	84378
Symmetry	C3	C3
Box size (pix)	320	320
Resolution (Å) (FSC0.143)*	3.0	2.9
Refinement (Phenix)		
Protein residues	999	1472
Chimera CC	0.77	0.63
R.m.s. deviations		
Bond lengths (Å)	0.01	0.01
Bond angles (°)	1.06	1.24
Validation		
Molprobity score	1.71	1.87
Clash score	4.4	6.0
Favored rotamers (%)	99.4	97.2
Ramachandran		
Favored regions (%)	91.6	90.3
Disallowed regions (%)	0.41	0.6

* Resolutions are reported according to the FSC 0.143 gold-standard criterion

Supplementary Table S5: PCA Cluster PDBs

Yellow	Orange	Green	Blue	Red	Black
6b0n	5u7o	6okp	6osy	*F14*	*F14Vt8*
5fyj	4zmj	5um8	6n1w	6n1v	6uda
6nnf	6mco	5cjx	6nc3	6orn	6orq
5v8m	6muf		6nf2		6orp
6ck9	5t3z		6cdi		6oro
5fyk	6mug		6mph		
6nm6	5d9q		6mpg		
6mtn	6ch8		6ot1		
5cez	4tvp		6cde		
6nnj	5i8h		6cuf		
6de7	5utf		6cue		
5aco	5t3x				
6ohy	6ch7				
6ieq					
6mtj					
6mu7					
6mu8					
6mu6					
5v7j					
5v8l					
6mdt					
5fyl					
5uty					
5u7m					

Supplementary Table 6: Cell Surface Expressed Trimer - Percentage of Cells Binding

	Control	BG505	BG505 DS	BG505 F14/Vt8	BG505 F14	BG505 Vt8
**N6**	0.3 ± 0.2	45.3 ± 8.1	41.0 ± 5.8	40.3 ± 3.7	53.9 ± 12.0	44.4 ± 5.0
**CH01**	0.2 ± 0.1	32.8 ± 2.6	20.8 ± 9.7	22.1 ± 11.9	37.7 ± 12.4	30.2 ± 3.0
**PGT125**	0.2 ± 0.1	47.1 ± 6.6	38.2 ± 9.1	37.9 ± 5.2	54.7 ± 10.1	43.2 ± 10.5
**PGT145**	0.3 ± 0.2	46.0 ± 4.6	35.4 ± 3.0	34.8 ± 2.6	48.8 ± 11.9	38.2 ± 4.5
**17B**	0.3 ± 0.1	9.0 ± 3.6	0.6 ± 0.2	1.2 ± 0.9	2.7 ± 1.0	0.8 ± 0.5
**19B**	0.2 ± 0.2	17.1 ± 8.2	16.8 ± 1.7	8.0 ± 4.8	23.5 ± 8.4	8.0 ± 5.4
**17B + sCD4**	0.2 ± 0.1	25.9 ± 7.1	1.3 ± 0.2	0.7 ± 0.3	3.7 ± 1.2	0.9 ± 0.5
**19B + sCD4**	0.2 ± 0.1	25.3 ± 8.9	16.9 ± 8.3	9.0 ± 5.5	23.6 ± 8.0	9.0 ± 6.4
**17B + CD4-Ig**	0.2 ± 0.1	32.9 ± 6.5	0.9 ± 0.3	0.8 ± 0.6	8.2 ± 0.8	9.5 ± 1.1
**19B + CD4-Ig**	0.3 ± 0.2	38.3 ± 8.2	15.0 ± 7.1	9.5 ± 5.6	27.0 ± 7.1	21.8 ± 5.5

* Values reported as the average of three experiments with standard deviations

Supplementary Table 7: Cell Surface Expressed Trimer - MFI

	Control	BG505	BG505 DS	BG505 F14/Vt8	BG505 F14	BG505 Vt8
**N6**	39.9 ± 14.1	457.3 ± 137.9	338.0 ± 72.6	341.3 ± 43.2	568.0 ± 152.6	378.3 ± 73.1
**17B**	34.1 ± 11.5	103.4 ± 38.6	49.2 ± 24.2	51.6 ± 28.0	60.0 ± 29.0	51.0 ± 25.0
**19B**	33.1 ± 11.5	211.3 ± 73.2	198.3 ± 67.9	109.3 ± 39.0	265.0 ± 84.2	103.8 ± 45.4
**17B + sCD4**	33.7 ± 11.1	244.7 ± 65.0	51.6 ± 27.1	48.8 ± 24.1	67.8 ± 34.0	48.4 ± 23.4
**19B + sCD4**	33.5 ± 11.4	306.7 ± 83.0	194.0 ± 63.0	115.2 ± 37.7	255.0 ± 88.4	114.8 ± 53.6
**17B + CD4-Ig**	42.5 ± 25.1	400.0 ± 115.2	50.2 ± 23.9	50.3 ± 28.4	102.4 ± 28.9	90.8 ± 29.1
**19B + CD4-Ig**	35.5 ± 13.1	531.3 ± 134.1	176.3 ± 62.7	119.5 ± 45.9	269.7 ± 63.2	190.7 ± 43.1

* Values reported as the average of three experiments with standard deviations

Supplementary Table 8: smFRET Statistics

gp160	BG505	BG505 + 12xCD4	BG505 F14/Vt8	BG505 F14/Vt8 + 12xCD4
**State 1**	46 ± 7	20 ± 8	49 ± 9	43 ± 7
**State 2**	26 ± 8	30 ± 11	20 ± 9	22 ± 11
**State 3**	28 ± 10	50 ± 13	31 ± 12	35 ± 12

* Values correspond to the means and standard deviations

Supplementary Figure 1

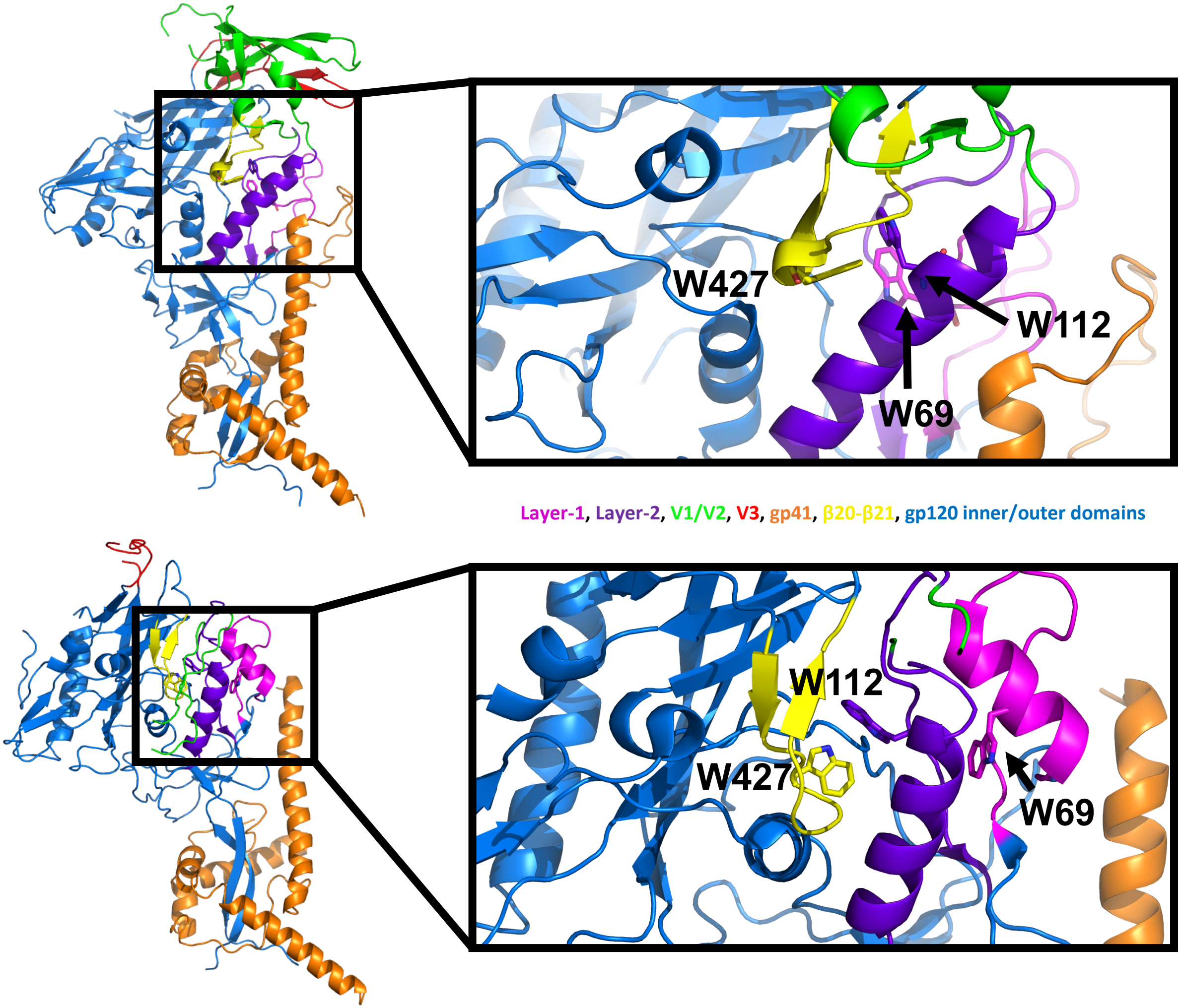

Supplementary Figure 1. *Structure of a closed and open state HIV-1 Env gp140 SOSIP protomer.* ***(A) (left)*** A closed state SOSIP gp140 protomer (PDB ID 5CEZ) identifying key structural elements. ***(right)*** Elements of the closed state allosteric network including β20-β21 (yellow), layer-1/2 (pink/purple), V1/2/3 (green/red) and tryptophans 69, 112 and 427. ***(B) (left)*** An open state SOSIP gp140 protomer (PDB ID 5VN3) identifying key structural elements. ***(right)*** Elements of the open state allosteric network including β20-β21 (yellow), layer-1/2 (pink/purple), V1/2/3 (green/red) and tryptophans 69, 112 and 427.

Supplementary Figure 2

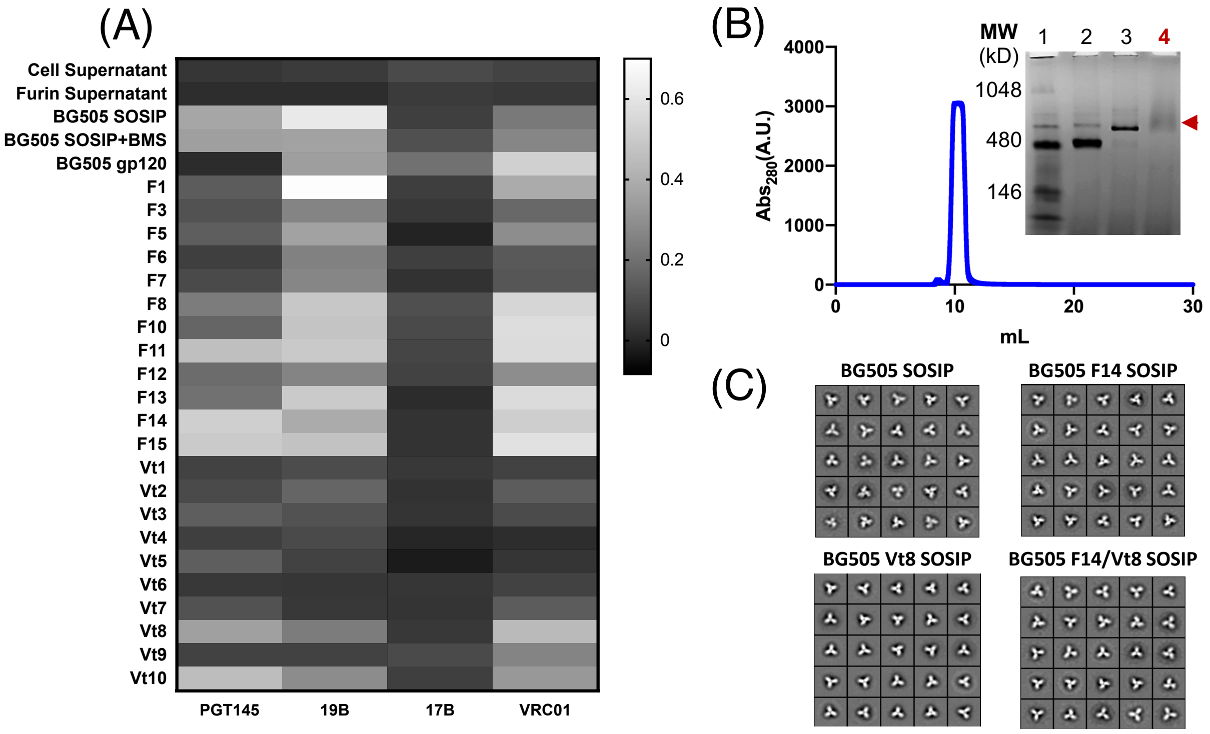

Supplementary Figure 2. *BG505 SOSIP Mutant Screening Results* ***(A)*** Transfection cell supernatant BLI heatmap for F-series, Vt-series, SOSIP, gp120, BMS-626529-bound BG505 SOSIP, and controls binding to PGT145, 19B, 17B, and VRC01. Results plotted as mean response (nm) from triplicate transfection supernatant screening. ***(B)*** *(graph)* Representative size exclusion chromatogram from the BG505 F14 SOSIP purification post PGT145 affinity column purification. *(inset)* Representative non-reducing SDS-PAGE gel for the BG505 SOSIP.664 mutants. Lanes 1-3 include protein marker, Ferritin, and Thyroglobulin, respectively. Lane 4 contains the SOSIP trimer. ***(C)*** Negative-stain EM two-dimensional class averages for purified BG505, F14, Vt8, and F14/Vt8 SOSIPs.

Supplementary Figure 3

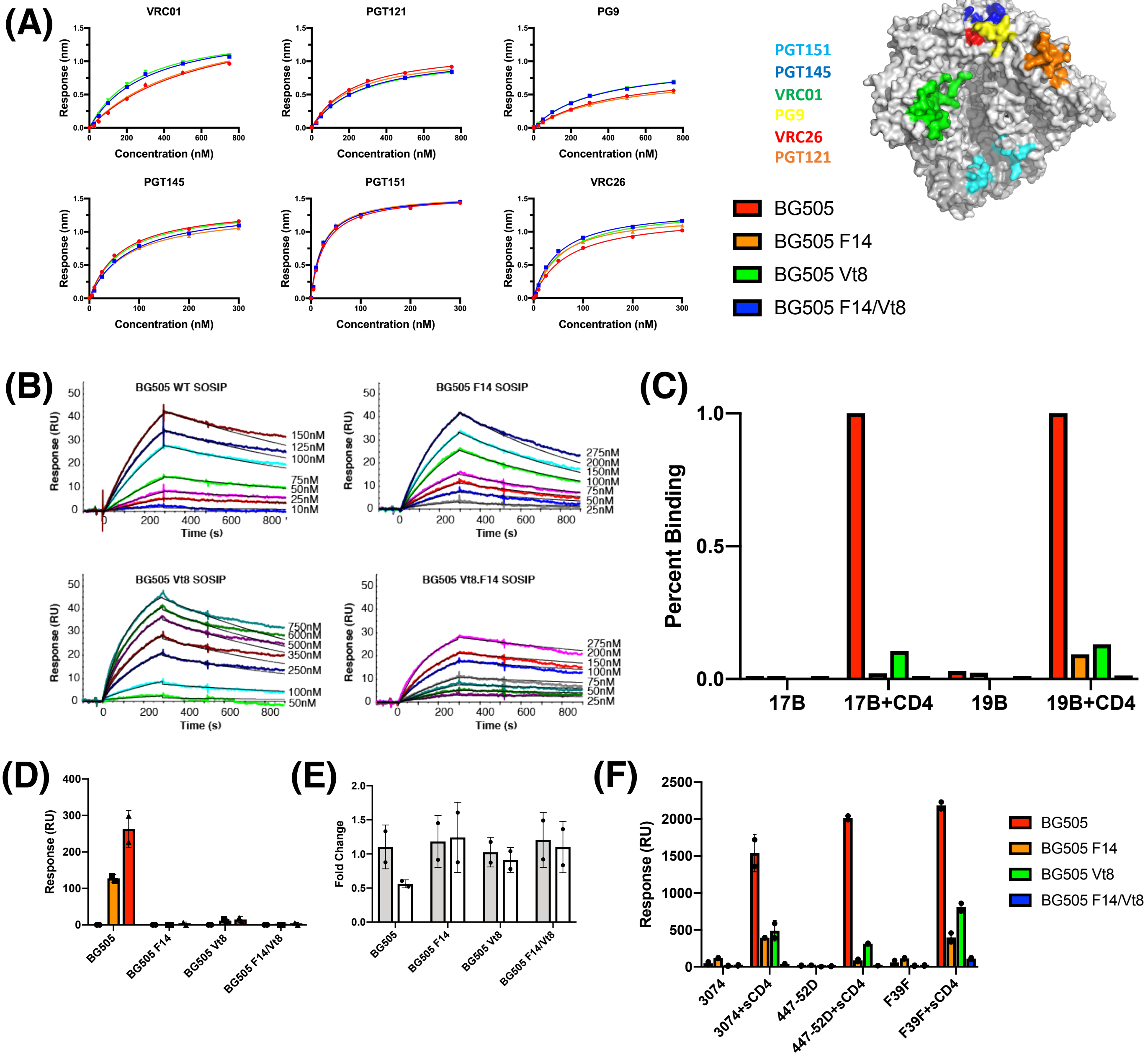

Supplemental Figure 3. *BG505 SOSIP Mutant bnAb Binding, CD4 binding, and CD4 Triggering* ***(A)*** *(left)* Dose response curves for binding of VRC01, PGT121, PG9, PGT145, PGT151, and VRC26 to the BG505, F14, Vt8, and F14/Vt8 SOSIPs. Results plotted as mean response (nm) from duplicate experiments (mean standard error is within plotted points). Lines represent one-site specific fitting of BLI binding data in Prism. *(right*) Surface representation of BG505 SOSIP with bnAb epitopes highlighted. ***(B)*** Representative SPR titrations for BG505, F14, Vt8, and F14/Vt8 SOSIP binding to eCD4-Ig. ***(C)*** Representative CD4 triggering results for BG505 (red), F14 (orange), Vt8 (Vt8), and F14/Vt8 (blue) SOSIP. Responses are normalized to BG505 SOSIP. ***(D)*** Response values for 17B interaction with BG505, F14, Vt8, and F14/Vt8 SOSIP alone (black) or incubated with sCD4 for either 30 minutes (orange) or 20 hours (red). ***E)*** Response ratio for PGT145 interaction with BG505, F14, Vt8, and F14/Vt8 SOSIP incubated with sCD4 for either 30 minutes (grey) or 20 hours (white) relative to the relevant unliganded trimer. ***F)*** Response values for V3 targeting 3074, 447-52D, and F39F interaction with BG505, F14, Vt8, and F14/Vt8 SOSIP in the absence and presence of sCD4. (D-F) N=2, error bars represent the standard deviation of the mean.

Supplementary Figure 4

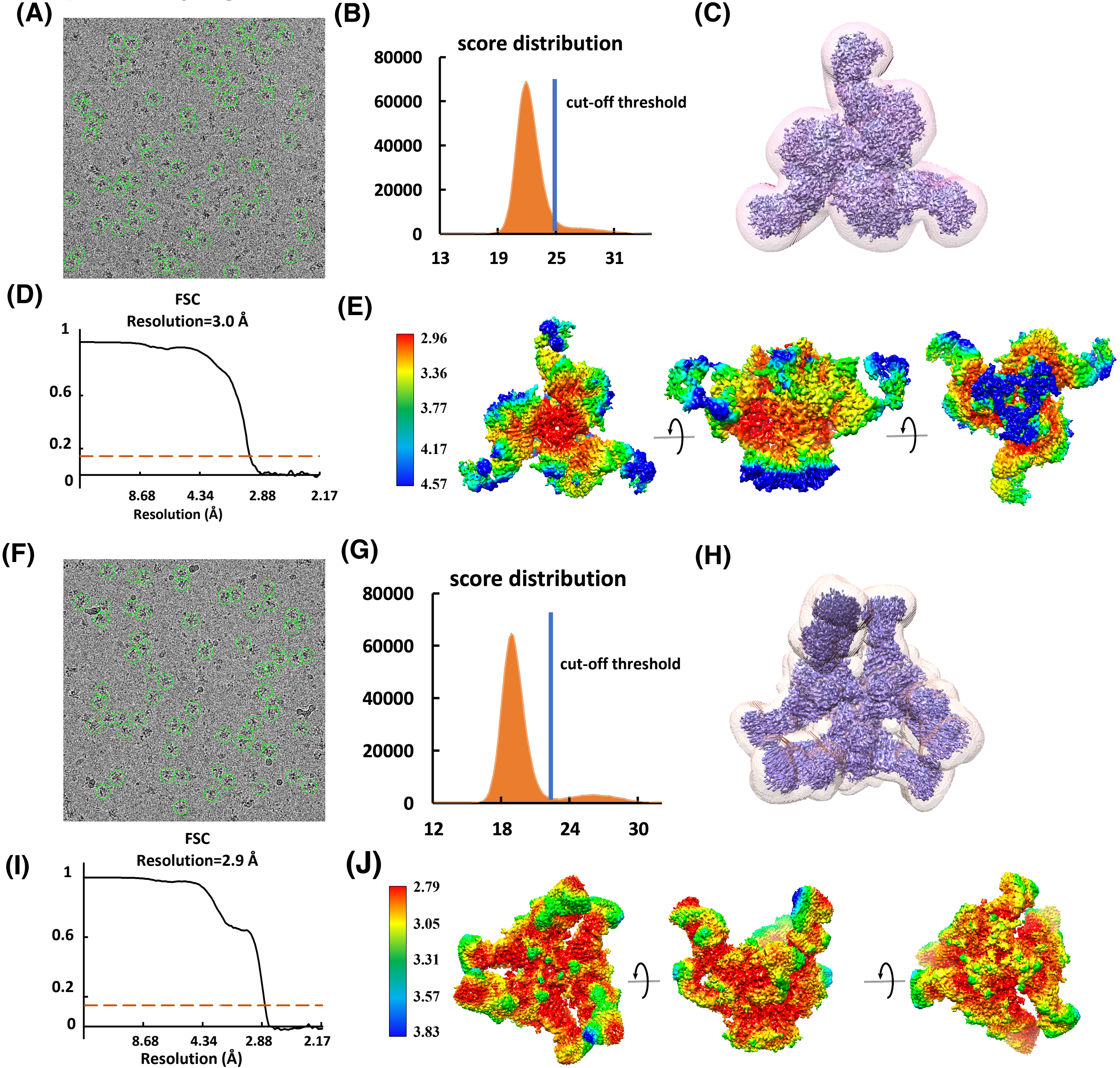

Supplementary Figure 4

Supplementary Figure 4. *Cryo-EM data analysis for BG505 F14 and F14/Vt8 SOSIP datasets.* **(A,F)** Representative micrographs showing particles selected for refinement from BG505-F14-SOSIP **(A)**, and BG505-F14/Vt8-SOSIP **(F)** datasets. **(B,G)** Distribution of cisTEM score values assigned to particles extracted from datasets BG505-F14-SOSIP **(B)**, and BG505-F14/Vt8-SOSIP **(G)**. **(C,H)** Cryo-EM map (solid) with superimposed shape mask (transparent) used during 3D refinement for BG505-F14-SOSIP **(C)**, and BG505-F14/Vt8-SOSIP **(H)** datasets. **(D,I)** Fourier Shell Correlation curves between half-maps showing estimated resolution according to the 0.143-cutoff criteria (dashed line) for BG505-F14-SOSIP **(D)** and BG505-F14/Vt8-SOSIP **(I)** reconstructions. **(E,J)** Local map resolution determined using RELION-3.0 for reconstructions of BG505-F14-SOSIP **(E)** and BG505-F14/Vt8-SOSIP **(J)**.

Supplementary Figure 5

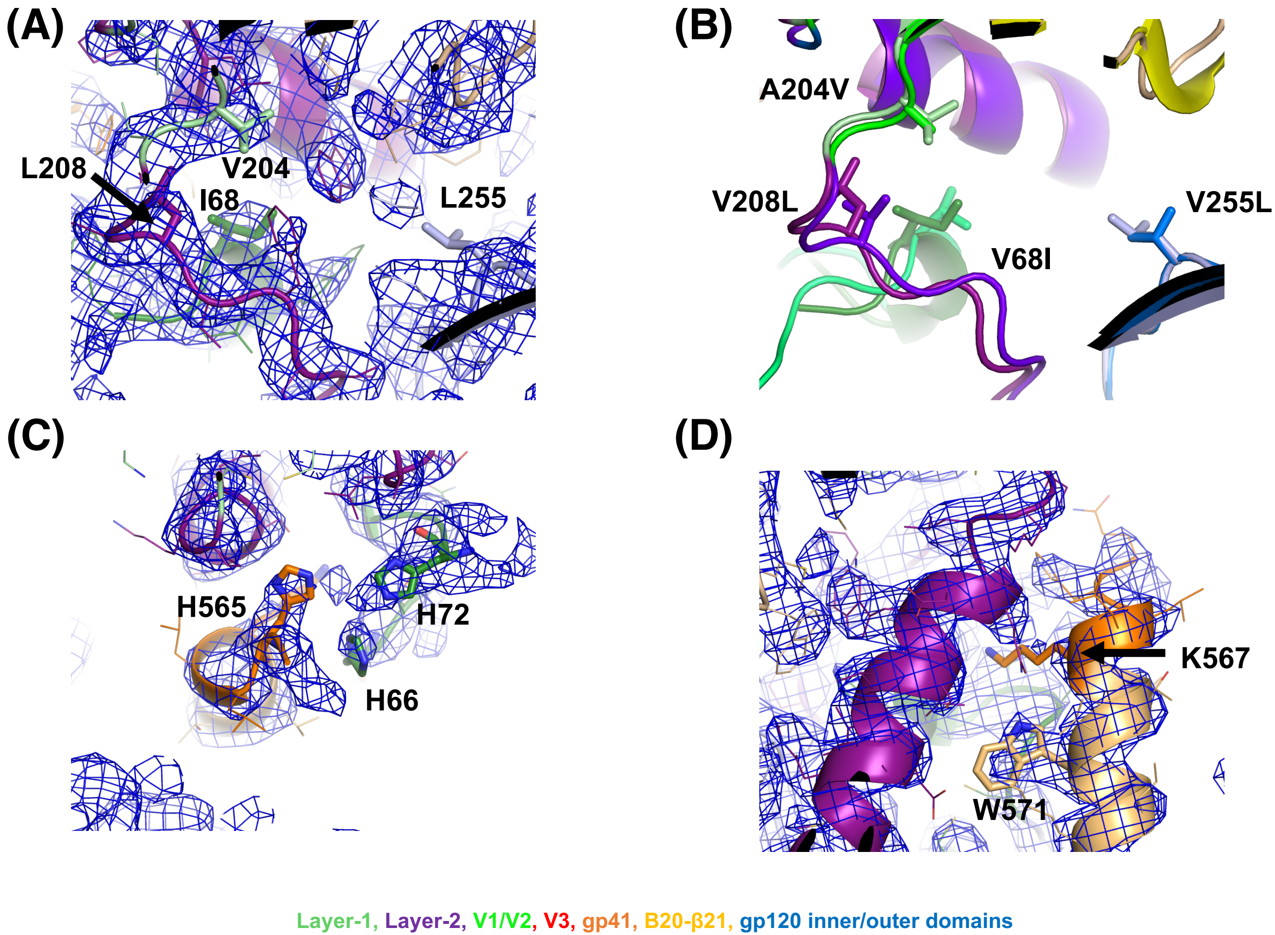

Supplementary Figure 5. *Cryo-EM fit of BG505 F14 mutations.* **(A)** Cryo-EM map of the BG505 F14 construct with fitted coordinates depicting the F14 mutation sites. **(B)** Alignment of the BG505 F14 SOSIP gp120 with the BG505 SOSIP (PDB ID 5CEZ) gp120 showing the relative positions of the F14 mutations. ***(C)*** Cryo-EM map (mesh) depicting H66, H72, and H565. ***(D)*** Cryo-EM map (mesh) depicting position of gp41 residues K567 and W571.

Supplementary Figure 6

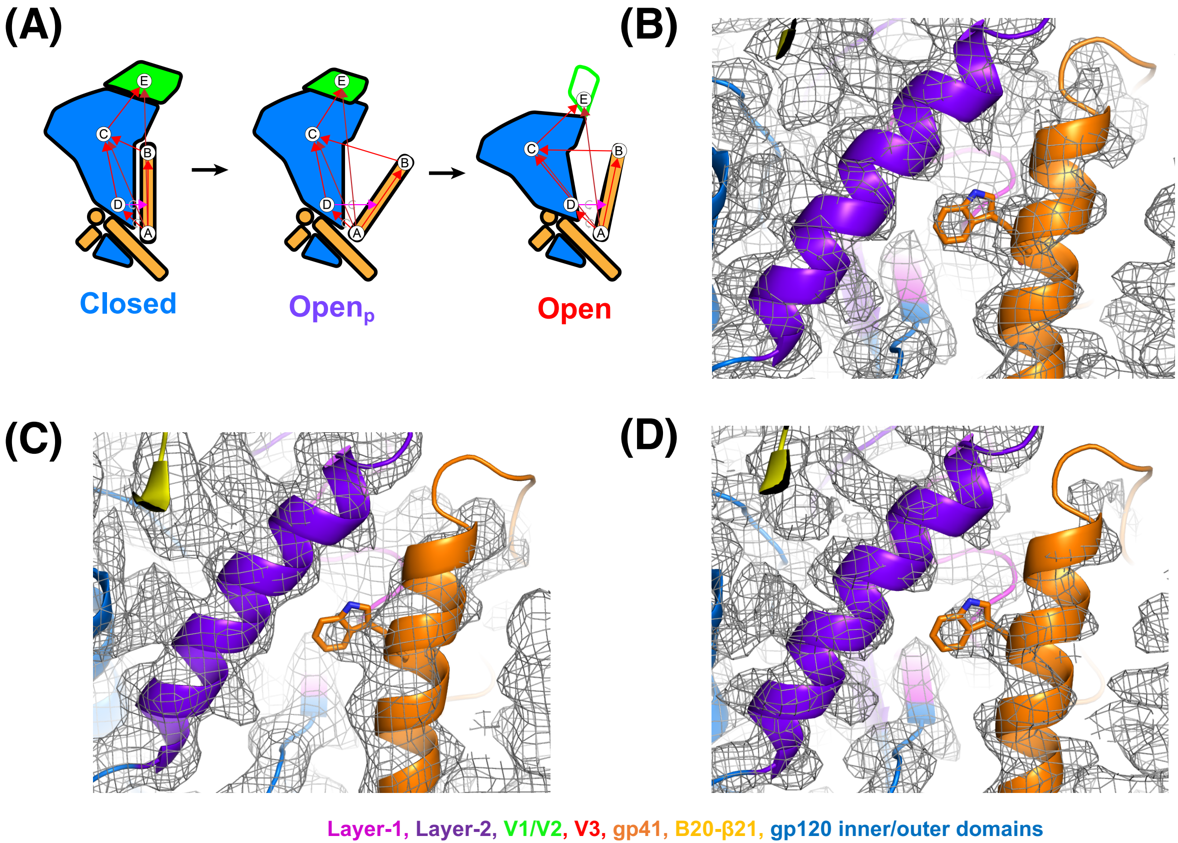

Supplementary Figure 6. *Vectors based analysis and cryo-EM fit of BG505 F14/Vt8 SOSIP coordinates to vFP bound BG505 DS SOSIP cryo-EM maps.* ***(A)*** Cartoon representations of gp120 (blue), gp120 V1/V2 region (green), and gp41 (yellow) in the closed, partially open, and open states. Letters indicate location of centroids with arrows depicting vectors between centroids. The reduced size of the V1/V2 region in the partially open state represents rearrangement in this region while the green outline of V1/V2 in the open state indicates complete dissociation and disorder. ***(B)*** Fit of the BG505 F14/Vt8 coordinates into the vFP20.01 bound BG505 DS SOSIP cryo-EM map (EMD-*7459)* ***(C)*** Fit of the BG505 F14/Vt8 coordinates into the vFP16.02 bound BG505 DS SOSIP cryo-EM map (EMD-*7460)* ***(D)*** Fit of the BG505 F14/Vt8 coordinates into the vFP1.01 bound BG505 DS SOSIP cryo-EM map (EMD-*7622)*

Supplementary Figure 7

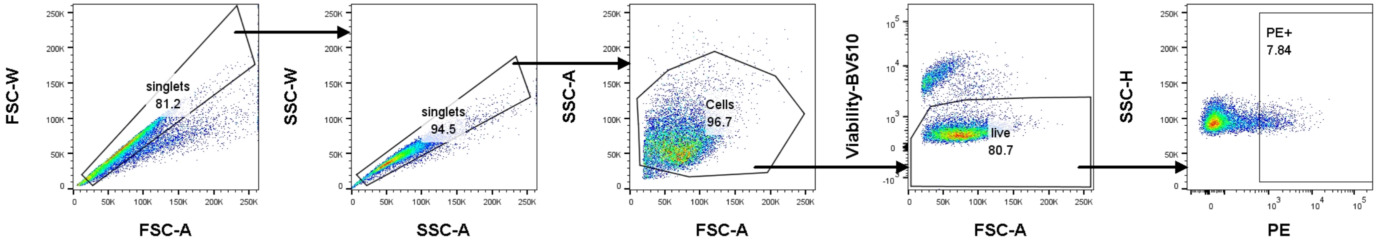

Supplementary Figure 7. ***F****low cytometry gating strategy.* Example data depicting the gating strategy used to assess Env cell surface expressed gp160 trimer binding to bnAbs and non-bnAbs.

Supplementary Figure 8

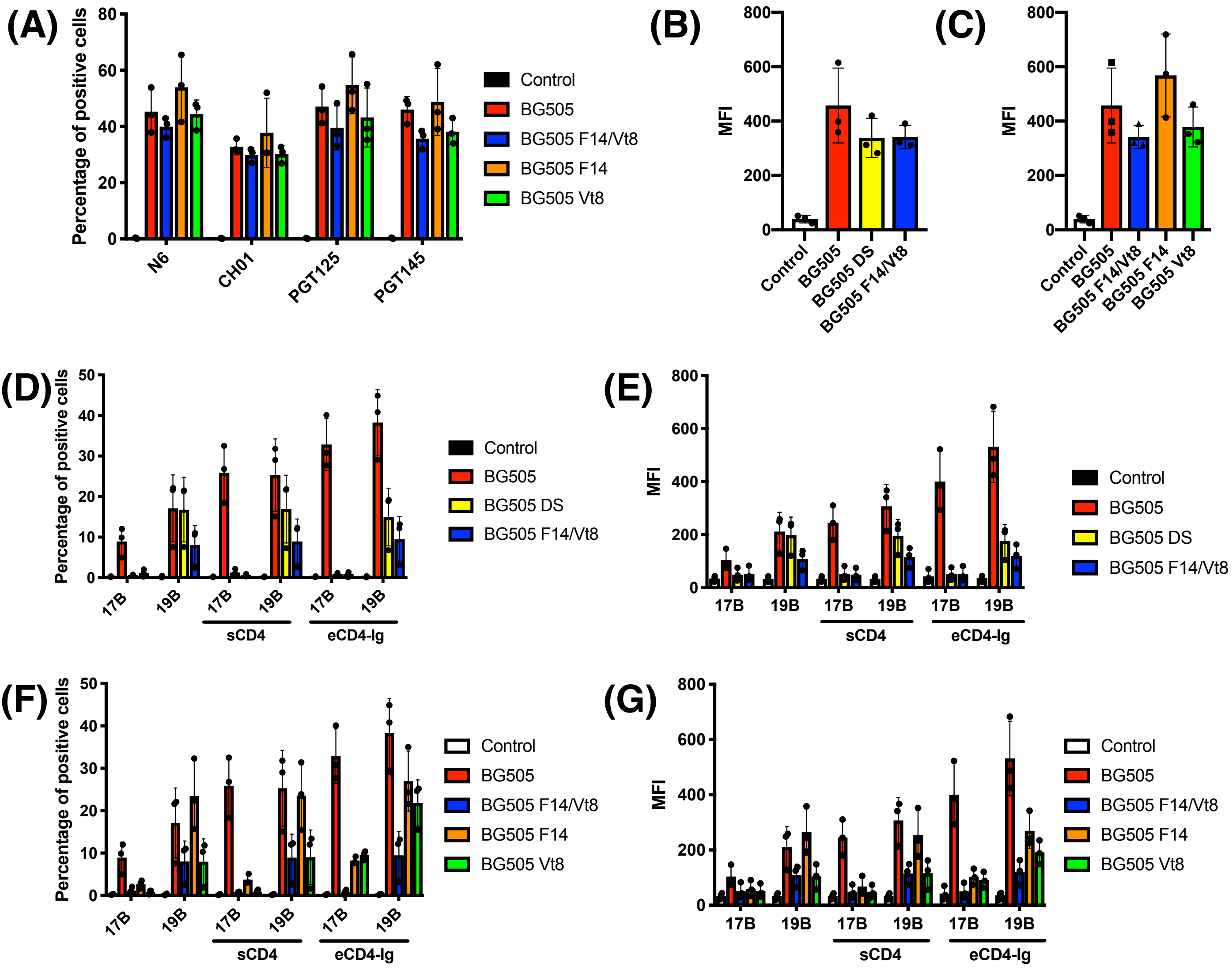

Supplementary Figure 8

Supplementary Figure 8. *Antigenicity and triggering of BG505 gp160 construct cell-surface expressed trimer.* ***(A)*** Percentage of cells positive for binding of bnAbs N6, CH01, PGT125, and PGT145 to 293F cell surface expressed gp160 BG505, BG505 F14/Vt8, BG505 F14, and BG505 Vt8 SOSIP trimers. ***(B)*** MFI data for binding of N6 to gp160 BG505, BG505 DS, and BG505 F14/Vt8 SOSIP trimers. ***(C)*** MFI data for binding of N6 to gp160 BG505, BG505 F14/Vt8, BG505 F14, and BG505 Vt8 SOSIP trimers. ***(D)*** Percentage of cells positive for binding of non-bnAbs 17B and 19B to 293F cell surface expressed BG505, BG505 DS, and BG505 F14/Vt8 SOSIP trimers in the presence and absence of sCD4 or CD4-Ig. ***(E)*** MFI data for binding of non-bnAbs 17B and 19B to 293F cell surface expressed BG505, BG505 DS, and BG505 F14/Vt8 SOSIP trimers in the presence and absence of sCD4 and CD4-Ig. ***(F)*** Percentage of cells positive for binding of non-bnAbs 17B and 19B to 293F cell surface expressed BG505, BG505 F14/Vt8, BG505 F14, and BG505 Vt8 SOSIP trimers in the presence and absence of sCD4 or eCD4-Ig. ***(G)*** MFI data for binding of non-bnAbs 17B and 19B to 293F cell surface expressed BG505, BG505 F14/Vt8, BG505 F14, and BG505 Vt8 SOSIP trimers in the presence and absence of sCD4 and eCD4-Ig. (A-G) N=3, error bars represent the standard deviation of the mean.

Supplementary Figure 9

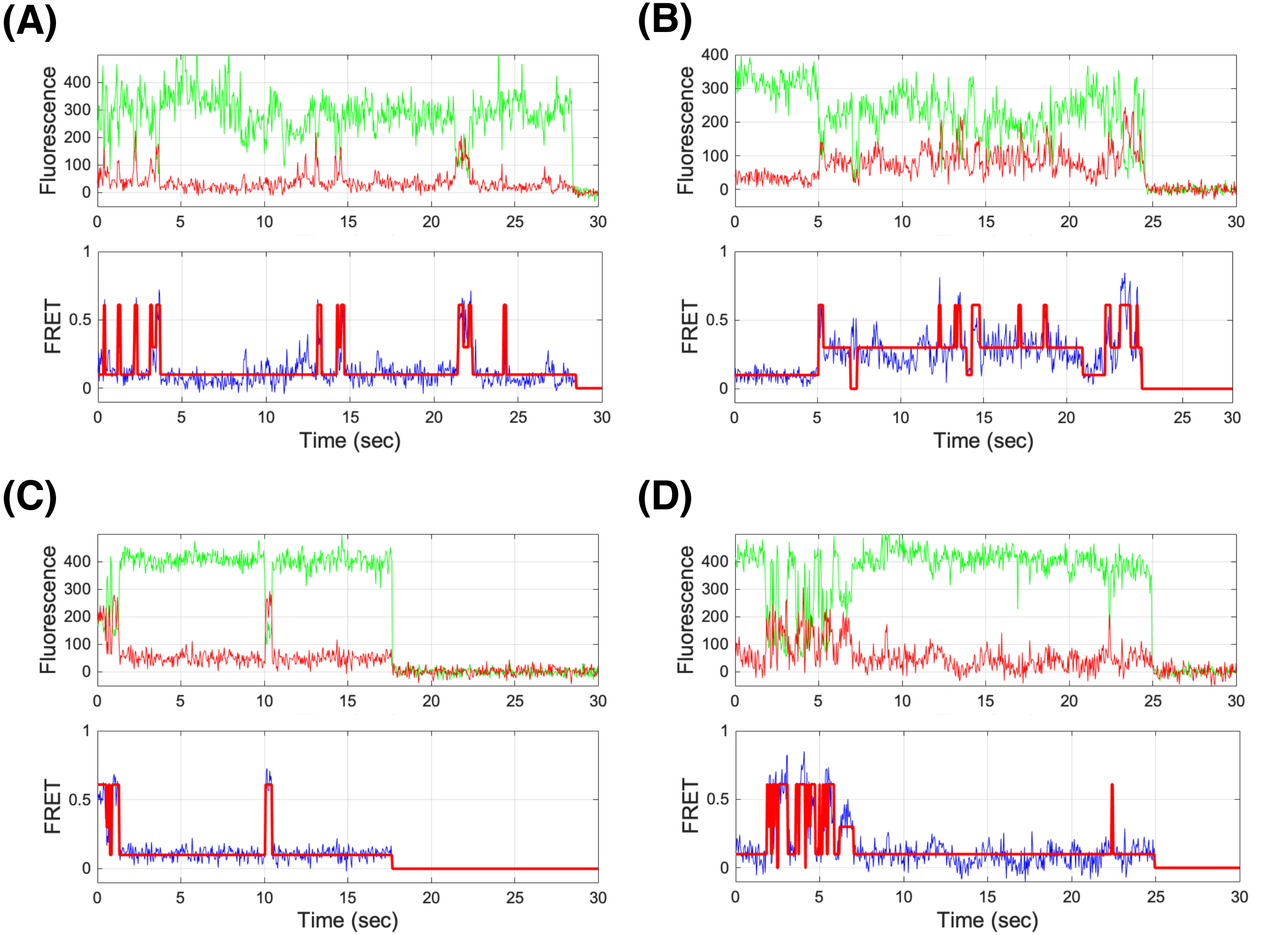

Supplementary Figure 9. *Representative fluorescence and FRET traces of both wild-type BG505 and BG505 F14/Vt8 virus Env in the absence and presence of 12xCD4* (Upper: Cy3 in green, Cy5 in red; Lower: resulting FRET in blue, HMM idealization in red) **(A)** Results for the BG505 Env. **(B)** Results for the BG505 Env in the presence of 12xCD4. **(C)** Results for the BG505 F14/Vt8 mutant Env. **(D)** Results for the BG505 F14/Vt8 Env mutant in the presence of 12xCD4.
