# Supplementary material for: Disruption of the HIV-1 Envelope allosteric network blocks CD4-induced rearrangements
Source: Nat Commun. 2020 Jan 24;11:520. doi: 10.1038/s41467-019-14196-w (PMC6981184; doi:10.1038/s41467-019-14196-w)
Supplement: Supplementary file 2 — Reporting Summary [file 41467_2019_14196_MOESM2_ESM.pdf]

nature research

Corresponding author(s): Rory Henderson, Priyamvada Acharya, S. Munir Alam

Last updated by author(s): Jun 24, 2019

Reporting Summary

Nature Research wishes to improve the reproducibility of the work that we publish. This form provides structure for consistency and transparency in reporting. For further information on Nature Research policies, see Authors & Referees and the Editorial Policy Checklist .

Statistics

For all statistical analyses, confirm that the following items are present in the figure legend, table legend, main text, or Methods section.

n/a	Confirmed	
☒	☐	The exact sample size (n) for each experimental group/condition, given as a discrete number and unit of measurement
☒	☐	A statement on whether measurements were taken from distinct samples or whether the same sample was measured repeatedly
☒	☐	The statistical test(s) used AND whether they are one- or two-sided<br>*Only common tests should be described solely by name; describe more complex techniques in the Methods section.*
☒	☐	A description of all covariates tested
☒	☐	A description of any assumptions or corrections, such as tests of normality and adjustment for multiple comparisons
☒	☐	A full description of the statistical parameters including central tendency (e.g. means) or other basic estimates (e.g. regression coefficient) AND variation (e.g. standard deviation) or associated estimates of uncertainty (e.g. confidence intervals)
☒	☐	For null hypothesis testing, the test statistic (e.g. F, t, r) with confidence intervals, effect sizes, degrees of freedom and P value noted<br>*Give P values as exact values whenever suitable.*
☒	☐	For Bayesian analysis, information on the choice of priors and Markov chain Monte Carlo settings
☒	☐	For hierarchical and complex designs, identification of the appropriate level for tests and full reporting of outcomes
☒	☐	Estimates of effect sizes (e.g. Cohen's d, Pearson's r), indicating how they were calculated

Our web collection on statistics for biologists contains articles on many of the points above.

Software and code

Policy information about availability of computer code

Data collection	FACSDIVA, EPU, LabView, Mathworks
Data analysis	Pymol, VMD, Octet Data Analysis 10.0, BIAevaluation 4.1, NanoAnalyze, cryoSPARC, UNBLUR, CTFFIND4, cisTEM, EMAN2, UCSF Chimera, Coot, MolProbity, Phenix, EMRinger, R,

For manuscripts utilizing custom algorithms or software that are central to the research but not yet described in published literature, software must be made available to editors/reviewers. We strongly encourage code deposition in a community repository (e.g. GitHub). See the Nature Research guidelines for submitting code & software for further information.

Data

Policy information about availability of data

All manuscripts must include a data availability statement. This statement should provide the following information, where applicable:

- Accession codes, unique identifiers, or web links for publicly available datasets
- A list of figures that have associated raw data
- A description of any restrictions on data availability

The datasets generated and/or analyzed during the current study are available from the corresponding author on reasonable request.

Field-specific reporting

Please select the one below that is the best fit for your research. If you are not sure, read the appropriate sections before making your selection.

☒ Life sciences ☐ Behavioural & social sciences ☐ Ecological, evolutionary & environmental sciences

Life sciences study design

All studies must disclose on these points even when the disclosure is negative.

Sample size	n/a
Data exclusions	n/a
Replication	Data are reported as the mean and standard deviation from a minimum of two replicate measurements unless otherwise noted (Tmax, SPR, BLI data, Flow Cytometry).
Randomization	n/a
Blinding	n/a

Reporting for specific materials, systems and methods

We require information from authors about some types of materials, experimental systems and methods used in many studies. Here, indicate whether each material, system or method listed is relevant to your study. If you are not sure if a list item applies to your research, read the appropriate section before selecting a response.

Materials & experimental systems

n/a	Involved in the study	
☐	☒	Antibodies
☐	☒	Eukaryotic cell lines
☒	☐	Palaeontology
☒	☐	Animals and other organisms
☒	☐	Human research participants
☒	☐	Clinical data

Methods

n/a	Involved in the study	
☒	☐	ChIP-seq
☐	☒	Flow cytometry
☒	☐	MRI-based neuroimaging

Antibodies

Antibodies used	All antibodies were produced in-house at the Duke Human Vaccine Institute and expression and purification methods are referenced in the article. The following antibody was used in SPR assays to capture mAbs on the sensor surface- goat anti-human IgG antibody, Fc, Supplier - Millipore-Sigma, Catalogue # AP113.
Validation	The above antibody manufactured by Millipore-Sigma lists on their website that this antibody is validated for use in ELISA, IP, WB for the detection of human IgG.

Eukaryotic cell lines

Policy information about cell lines

Cell line source(s)	Freestyle 293 cells from Invitrogen was used to express Env gp120 and gp140 proteins
Authentication	Manufacturer of the cell line provides authentication information on their website
Mycoplasma contamination	Mycoplasma contamination screening is described on the manufacturer's website
Commonly misidentified lines (See ICLAC register)	n/a

Flow Cytometry

Plots

Confirm that:

- [x] The axis labels state the marker and fluorochrome used (e.g. CD4-FITC).
- [x] The axis scales are clearly visible. Include numbers along axes only for bottom left plot of group (a 'group' is an analysis of identical markers).
- [x] All plots are contour plots with outliers or pseudocolor plots.
- [x] A numerical value for number of cells or percentage (with statistics) is provided.

Methodology

Sample preparation	FreeStyle 293F cells (ThermoFisher, catalog #R79007) were transfected using a mixture of 1ug HIV Env gp160s DNA in 75ul jetPRIME buffer (Polyplus-transfection) with 2ul jetPRIME (Polyplus-transfection) following the manufacturer's protocol.
Instrument	Flow cytometric data were acquired on a LSRII
Software	Data collection was performed using the FACSDIVA software (BD Biosciences) and were analyzed with FlowJo software
Cell population abundance	For each sample, a total number of 17000~20000 events were recorded. Typically, 80% of total events are singlets, and 65~85% of the single cells are live. This resulted in about 10000 events (50~60% of total events) for PE+ analysis. 293F cells were used for transfection, so sample purity wasn't a issue in these assays.
Gating strategy	Single cells were gated with FSC-A/FSC-W and SSC-A/SSC-W. Then, cells were gated with FSC-A/SSC-A. Following this, FSC-A/Aqua L_D-BV421 was used to gate Aqua- live cells. Live cells were then gated on PE. PE+ gate was decided by non-transfected (mock) group.

- [x] Tick this box to confirm that a figure exemplifying the gating strategy is provided in the Supplementary Information.
